# Association of Cerebral Networks in Resting State with Sexual Preference of Homosexual Men: A Study of Regional Homogeneity and Functional Connectivity

**DOI:** 10.1371/journal.pone.0059426

**Published:** 2013-03-21

**Authors:** Shaohua Hu, Dongrong Xu, Bradley Peterson, Qidong Wang, Xiaofu He, Jianbo Hu, Xiaojun Xu, Ning Wei, Dan Long, Manli Huang, Weihua Zhou, Weijuan Xu, Minming Zhang, Yi Xu

**Affiliations:** 1 Department of Mental Health, First Affiliated Hospital, College of Medicine, Zhejiang University, Hangzhou, China; 2 MRI Unit, Department of Psychiatry, Columbia University and New York State Psychiatric Institute, New York, New York, United States of America; 3 Department of Radiology, First Affiliated Hospital, College of Medicine, Zhejiang University, Hangzhou, China; 4 Department of Radiology, Second Affiliated Hospital, College of Medicine, Zhejiang University, Hangzhou, China; Beijing Normal University, China

## Abstract

Recent imaging studies have shown that brain morphology and neural activity during sexual arousal differ between homosexual and heterosexual men. However, functional differences in neural networks at the resting state is unknown. The study is to characterize the association of homosexual preference with measures of regional homogeneity and functional connectivity in the resting state. Participants were 26 healthy homosexual men and 26 age-matched healthy heterosexual men in whom we collected echo planar magnetic resonance imaging data in the resting state. The sexual orientation was evaluated using the Kinsey Scale. We first assessed group differences in regional homogeneity and then, taking the identified differences as seed regions, we compared groups in measures of functional connectivity from those seeds. The behavioral significances of the differences in regional homogeneity and functional connectivity were assessed by examining their associations with Kinsey Scores. Homosexual participants showed significantly reduced regional homogeneity in the left inferior occipital gyrus, right middle occipital gyrus, right superior occipital gyrus, left cuneus, right precuneus, and increased regional homogeneity in rectal gyrus, bilateral midbrain, and left temporal lobe. Regional homogeneity correlated positively with Kinsey scores in the left inferior occipital gyrus. The homosexual group also showed reduced functional connectivity between left middle temporal gyrus, left supra-marginal gyrus, right cuneus and the seed region, i.e. left inferior occipital gyrus. Additionly, the connection between the left inferior occipital gyrus and right thalamus correlated positively with Kinsey scores. These differences in regional homogeneity and functional connectivity may contribute to a better understanding of the neural basis of male sexual orientation.

## Introduction

The neural basis of sexual orientation, which has been studied intensively for more than a century, has been hypothesized to relate to neuroendocrine influences [1], neurotransmitters [2], brain morphology [3]–[5], and genetic factors [6]. Imaging studies have reported reduced rightward asymmetries in overall volumes of the cerebral hemispheres in homosexual and heterosexual women compared with heterosexual men and homosexual women [7]. Witelson *et al*. [8] found the isthmus of the corpus callosum to be larger in homosexual compared with heterosexual men, suggesting that homosexual men have less marked functional asymmetry in the cortical regions that send their axons through the callosum [7]. Cross-sectional area of the anterior commissure was also reported larger in homosexual than heterosexual men [4].

Functional MRI studies have suggested that brain functioning differs in homosexual compared with heterosexual individuals. Paul *et al.* [9] demonstrated that brain responses of heterosexual males to heterosexual stimuli is similar to that of homosexual males exposed to homosexual stimuli, suggesting a uniform neural response to the object of sexual desire that is independent of sexual orientation. They also reported that despite equal ratings of sexual arousal, homosexual males and heterosexuasl women activated their hypothalamus less than did heterosexual men to visual erotic stimuli. When smelling pheromone-like compounds, both homosexual and heterosexual men activate the media preoptic area of the hypothalamus, but the magnitude of that activation depends on sexual preference [10]. In addition, visual face stimuli elicit much stronger activation of the dorsomedial thalamus and medial orbitofrontal cortex when the gender of the face matches a participant’s sexual preference than that of non-preferred face stimuli, regardless of the participant’s sexual orientation [11], findings confirmed in another study [12]. Another study reported that preference-related activity was greater in the amygdala of homosexual than heterosexual individuals [13]. Our prior study [14] found that the left angular gyrus, left caudate nucleus, and right pallidum were activated in homosexual men during visually evoked sexual arousal; in contrast, we found that heterosexual men showed no activation in these regions. However, heterosexual men showed activation in the bilateral lingual gyrus, right hippocampus, and right parahippocampal gyrus, areas that were not activated in homosexual men. Although Ponseti and colleagues [15] suggested that sexual orientation could be assessed by hemodynamic brain response to visual sexual stimuli, one study showed no difference in the pattern of activation between homosexual and heterosexual participants who were viewing faces of romantic partners of either the opposite or the same gender [16].

A prominent limitation common to all these studies is that perceptional and cognitive processes identified in task-related studies can be learned rather than innate [7] and therefore unlikely to represent the innate neural basis for sexual orientation. In addition, group differences in brain activity could be a cognitive or emotional response to the stimulus or performance on the task rather than a neural determinant of sexual orientation. Thus, it is important to assess group differences in brain functioning at rest to minimize these latter confounds of task and study design. In the study, we used a measure of regional homogeneity (ReHo) in brain activity to derive measures of local connectivity in brain functioning. Then, taking the identified differences as seed regions, we compared groups in measures of functional connectivity from those seeds. The ReHo method tests for local correlations in BOLD time series, using Kendall’s coefficient of concordance (KCC). KCC is based on time course correlations between a voxel and its neighbors. Mathematically, ReHo is comparable to the conventional functional connectivity. However, whereas functional connectivity tests correlations between distal voxels or regions of interest for BOLD time series, focusing on long-distance interregional connectivity, ReHo targets connectivity an the local level. The study is to characterize the association of homosexual preference with measures of ReHo and functional connectivity in the resting state.

## Methods

### Participants

The study was approved by the ethics committee of the First Affiliated Hospital, College of Medicine, Zhejiang University. Twenty-six healthy men with a self-identified homosexual preference and twenty-six healthy heterosexual men were recruited in the study. All the homosexual participants were recruited via local underground clubs of homosexuality. We presented to all participants the objectives and content of the study, and all provided written informed consent to participate.

We assessed the sexual orientation of each participant using the Kinsey Heterosexual-Homosexual Rating Scale [17], which ranges in values from 0 (denoting exclusively heterosexual orientation) to and 6 (for exclusively homosexual orientation), with a score of 3 indicating equally homosexual and heterosexual (bisexual). The ratings of all heterosexual participants totaled 0, and those of the homosexual participants ranged from 3 to 6 (Table 1). In a structured interview, we verified that all the participants were right-handed and had no history of substance abuse or major medical illness. All participants reported no symptoms of sexual dysfunction, gender identity disorder, paraphilia, or sexual offences.

**Table 1:** Demographic data and sexual preference characteristics for 26 homosexual and 26 heterosexual participants.

	Group				
Characteristic	Homosexual Participants		Heterosexual Participants		
	Mean	SD^b^	Mean	SD	P^c^
Age(years)	22.27	3.5	23.46	2.79	0.18
	N	%	N	%	
Kinsey Scale^a^					
Score 0	0	0	26	100	
Score 1	0	0	0	0	
Score 2	0	0	0	0	
Score 3	4	15.38	0	0	
Score 4	6	23.08	0	0	
Score 5	9	34.62	0	0	
Score 6	7	26.92	0	0	

a All heterosexual participants scored themselves as exclusive heterosexual, whereas none of the homosexual participants scored themselves as heterosexual but at least bisexual.

b SD: standard deviation,

c p: the age difference between two groups was no statistically significant.

### Data Acquisition

Magnetic Resonance Imaging (MRI) was performed on a 3.0-Tesla General Electric (GE) Signa Scanner using a standard GE quadrature head coil. Participants were equipped with ear plugs and their heads were padded with foam to reduce head motion. High resolution axial T1- and T2-weithed images were obtained to exclude the presence of silent lesions. Resting-state fMRI scans were performed using an echo planar imaging (EPI) sequence with the following parameters: repetition time = 2000 ms, echo time = 30 ms, flip angle = 90°, matrix = 64×64, field of view = 24×24 cm^2^, slice thickness = 3.0 mm, and gap = 0.8 mm. Each brain volume comprised 32 axial slices and each functional run contained 180 volumes. During fMRI scanning, all participants were instructed to relax with their eyes closed, without falling asleep and without thinking of anything in particular (confirmed by the participants immediately after the scan).

### ReHo Analysis

The fMRI dataset from each participant contained images acquired at 180 time points. The images were preprocessed using a toolkit (DPARSF, www.restfmri.net) as follows: The first ten time points of fMRI data were discarded to allow signal to reach equilibrium and participants to acclimate to the scanner noise. The remaining images were slice-time corrected, spatially aligned to the first image of each session for motion correction, and then spatially normalized using the SPM’s echo-planar imaging (EPI) template [18]. Each participant’s movement parameters were examined with the intention of excluding datasets with a maximal translation more than 2 mm or a maximal rotation more than 2 degrees in any direction, but as head movement for all participants was less than 1.5 mm and 1.5 degree, no dataset was excluded. DPARSF removed systematic drift or linear trend as well as low-frequency drift using a temporal filter (0.01 Hz<f <0.08 Hz) [19].

Kendall’s coefficient of concordance (KCC) [20] was used voxel-wise to measure similarity of the time series at a given voxel with its nearest neighboring voxels.
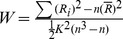
where, *W* is the KCC among given voxels, ranging from 0 to 1; *R_i_* is the sum rank of the *i*th time point; 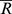 = ((*n* +1) *K*)/2 is the mean of the *R_i_*’s; *K* is the number of time series within a measured cluster; and *n* is the number of ranks (here, *n* = 170 time points). The KCC program was coded in MATLAB (The MathWorks, Inc., Natick, MA). Individual ReHo maps were generated by calculating KCC at each voxel, using a brain mask which was porvided by the DPARSF software. For standardization purposes, each individual ReHo map was divided by its own mean ReHo within the mask. Then, the data were smoothed using a Gaussian filter with a full width at half-maximum (FWHM) of 4 mm to reduce noise and residual difference in gyral anatomy. A second–level, random-effect, one-sample *t*-test (*P*<0.05, with multiple comparison correction) against the mean KCC (i.e. against 1) was performed. Then, a second-level random-effect two-sample *t*-test (*P*<0.05, corrected) was performed to compare the ReHo results from the two groups in the resting state in order to explore the effect of sexual orientation on ReHo. Threshold correction was performed using the AlphaSim program in AFNI (Parameters: FWHM = 4 mm, Cluster connection radius r (mm ) = 5.00, with a gray matter mask and resolution of 3 mm×3 mm×3 mm, p on individual voxel = 0.05, cluster size (voxels) = 85) which applies Monte Carlo stimulation to calculate the probability of false positive detection by considering both probability thresholding and cluster size at each voxel. Finally, to explore whether ReHo correlates with the degree of homosexual preference in the homosexual group, the correlation of ReHo with Kinsey Scale scores was assessed in the homosexual group at each voxel. Threshold correction was also performed using the AlphaSim program as above mentioned.

### Functional Connectivity Analysis

The images were preprocessed using a toolkit (REST, www.restfmri.net). Functional connectivity (FC) was examined using a method based on correlation with activity in a seed voxel or a seed region [21], [22]. The seeding areas selected for FC analysis were to be those where ReHo correlated significantly with the Kinsey scale for sexual preference. A reference time series for each seed was obtained by averaging the fMRI time series for all voxels within each of the seed areas. Then, the time series for all voxels were temporally band-pass filtered (0.01–0.08 Hz). A voxel-wise correlation analysis was conducted between the seed reference and the rest of the brain. By Fisher Z scores transformation using REST toolkit, individual r-value map was transformed into *z*-value map. For within-group analyses, individual *z*-value maps were analyzed with a random effect one-sample t-test to identify voxels showing a significant positive or negative correlation with the time series in the seed region, with correlations thresholded and corrected at p<0.05. For between-group analyses, two-sample t-tests compared *z*-value maps between the homosexual and heterosexual groups, with the significance threshold set at p<0.05 and corrected for multiple comparisons. To identify the association between functional connectivity and degree of homosexual preference in the homosexual group, Person’s correlation coefficient *r* was computed and then correlated with Kinsey Scale scores for the homosexual participants and thresholded at p<0.01 after correction for multiple comparisons from the theory of random Gaussian fields.

## Results

### ReHo Analysis

**Figure 1:**
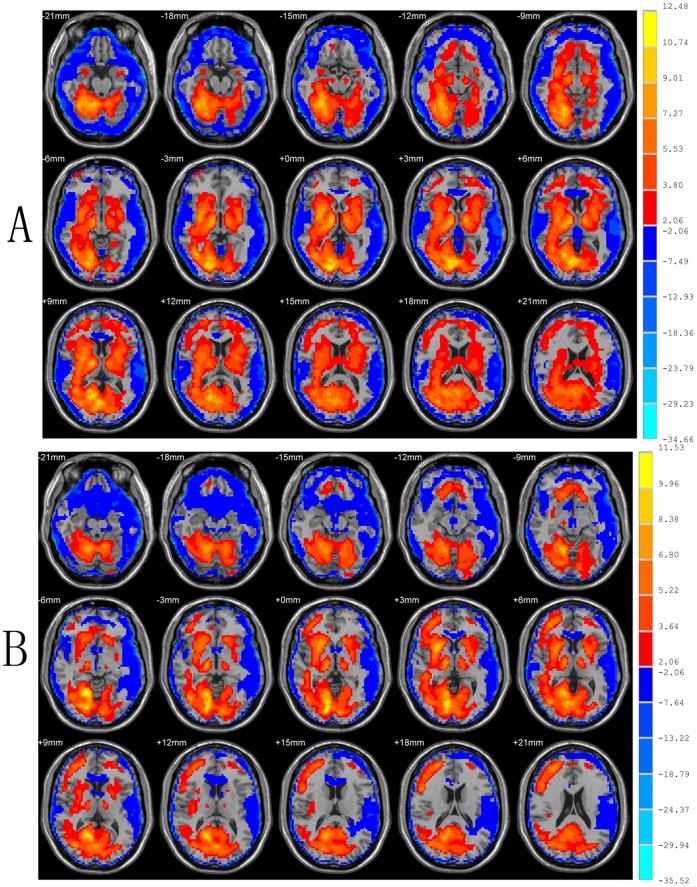
Regional homogeneity (ReHo) in the resting state, shown as maps of Kendall’s coefficient of concordance (KCC) within the homosexual group (A) and heterosexual group (B) (one-sample *t-*test; p<0.05, with multiple correction). T-sore bars are shown on the right. Hot and cold colors indicate higher or lower than the global mean, respectively.

ReHo values in the resting state within the homosexual and heterosexual groups are shown in Figure 1 (one-sample *t*-test; P<0.05, corrected for multiple comparisons). Across groups, the default mode network in common included left thalamus, right superior frontal gyrus, right middle frontal gyrus, and left precuneus (Fig. 1).

**Figure 2:**
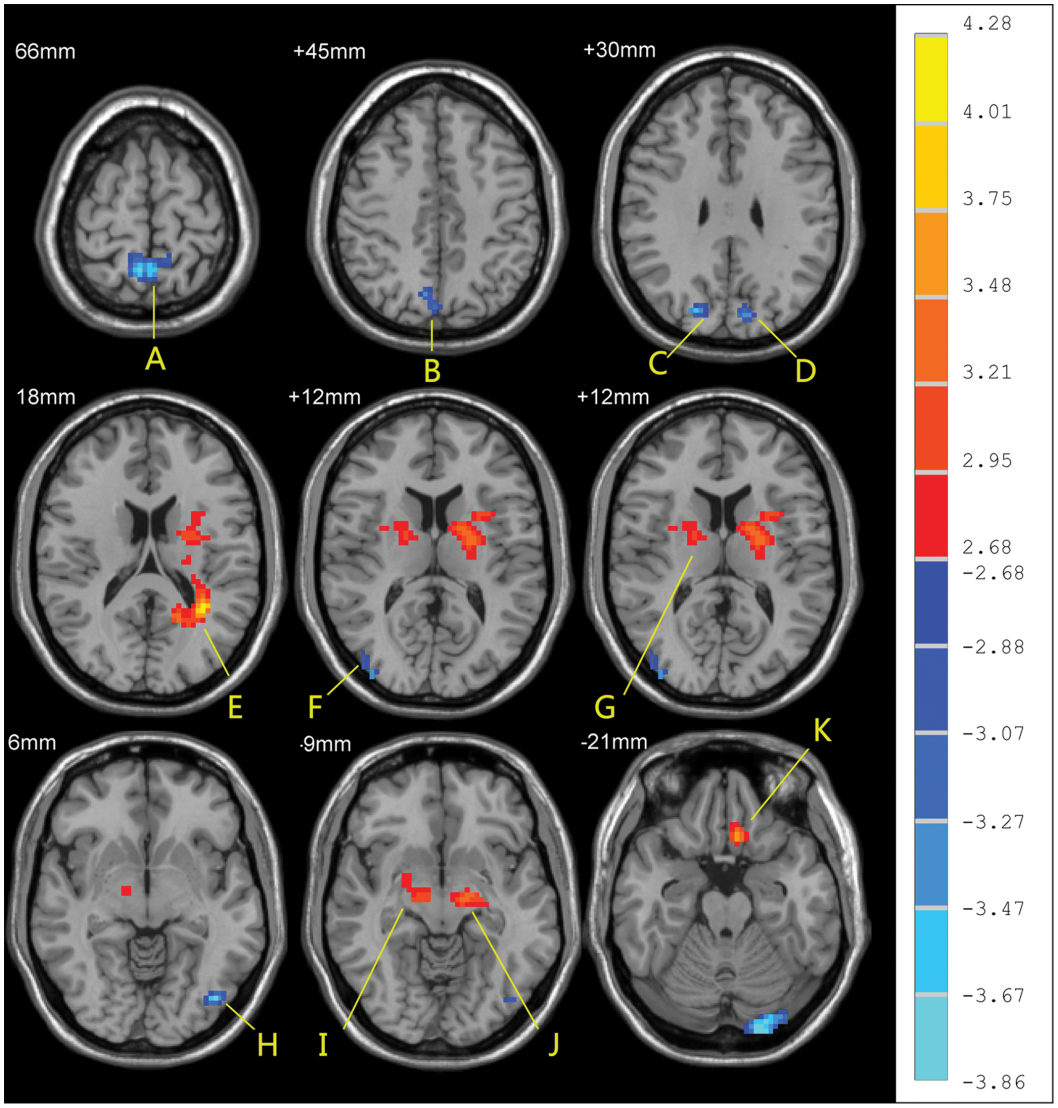
Results of ReHo shown as a comparison of KCC maps between homosexual and heterosexual groups in the resting state (two-sample *t*-test; p<0.05, corrected). T-score bars are shown on the right. Hot and cold colors indicate increased and decreased ReHo in homosexual group, respectively, compared with heterosexual group. The identified brain regions are the following: (A&B) right precuneus; (C) right superior occipital gyrus; (D) left cuneus; (E) left temporal lobe; (F) right middle occipital gyrus; (G) right extra-nuclear white matter; (H) left inferior occipital; (I) right midbrain; (J) left midbrain; (K) left rectal gyrus.

Compared with the heterosexual group, the homosexual group in the resting state showed significantly increased ReHo in the left rectal gyrus, bilateral midbrain, left temporal lobe and right extra-nuclear white matter, and decreased ReHo in left inferior occipital gyrus, right middle occipital gyrus, right superior occipital gyrus, left cuneus, right precuneus (two-sample *t* test, *P*<0.05, corrected; Figure 2, Table 2).

**Table 2:** Differences of ReHo between the homosexual and heterosexual groups in resting state (two-sample *t-*test, p<0.05, corrected).

Brain areas	Brodmann areas	Coordinates			t value	Cluster size
		x	y	z		
Left Rectal Gyrus		−6	24	−21	3.70	23
Left Midbrain		−15	−12	−9	3.37	26
Right Midbrain		12	−12	−9	3.18	30
Left Temporal Lobe		−33	−51	18	4.28	283
Right Extra-Nuclear White Matter		18	−3	12	2.96	27
Left Inferior Occipital Gyrus		−42	−78	−6	−3.72	27
Right Middle Occipital Gyrus	19	39	−90	12	−3.31	18
Left Cuneus		−9	−84	30	−3.43	20
Right Superior Occipital Gyrus	19	21	−81	30	−3.81	33
Right Precuneus		9	−69	45	−3.44	27
Right Precuneus	7	6	−54	66	−3.75	76

### Correlation between ReHo and Kinsey Scale Scores in Homosexual Group

**Figure 3:**
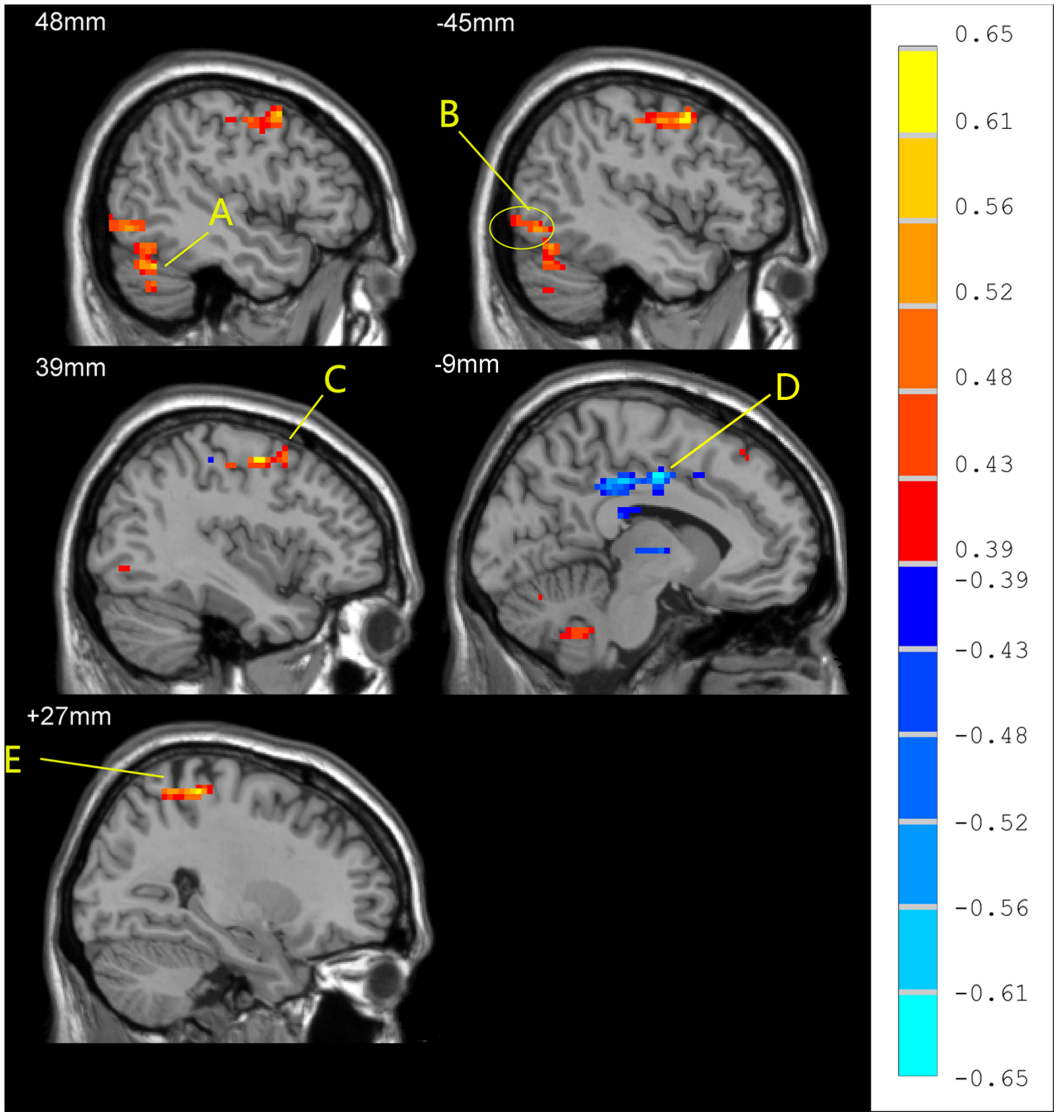
Significant correlations of ReHo in the resting state with in the homosexual group with Kinsey Scale scores (correlation analysis, p<0.05, corrected). R-value are shown on the right. Hot and cold colors indicate the positive and negative correlations, respectively. The identified brain regions are the following: (A) left Cerebelum; (B) left inferior occipital gyrus; the cluster circled was selected as seed for FC analysis; (C) left precentral cortex; (D) left supplementary motor area; (E) right postcentral cortex.

The correlation of ReHo at each voxel with scores on the Kinsey Scale in the homosexual group at resting state were significantly positive in left inferior occipital gyrus, left cerebellum, left precentral gyrus, and right postcentral gyrus, and significantly negative in left superior motor area and right superior parietal gyrus (P<0.05, corrected; Figure 3, Table 3).

**Table 3:** Brain Region showing significantly correlations between ReHo and the Kinsey Scale scores in homosexual group (correlation analysis, p<0.05, corrected).

Brian Region	Brodmann Area	Coordinates			r value	Cluster Size
		x	y	z		
Left Inferior Occipital Gyrus		−45	−72	−9	0.53	142
Cerebelum_Crus1_L		−48	−60	−30	0.56	104
Left Precentral cortex	6	−39	−3	51	0.63	124
Right Postcentral cortex	3	27	−36	60	0.59	155
Left Supplementary Motor Area		−9	−9	43	−0.63	755

### FC Analysis

**Figure 4:**
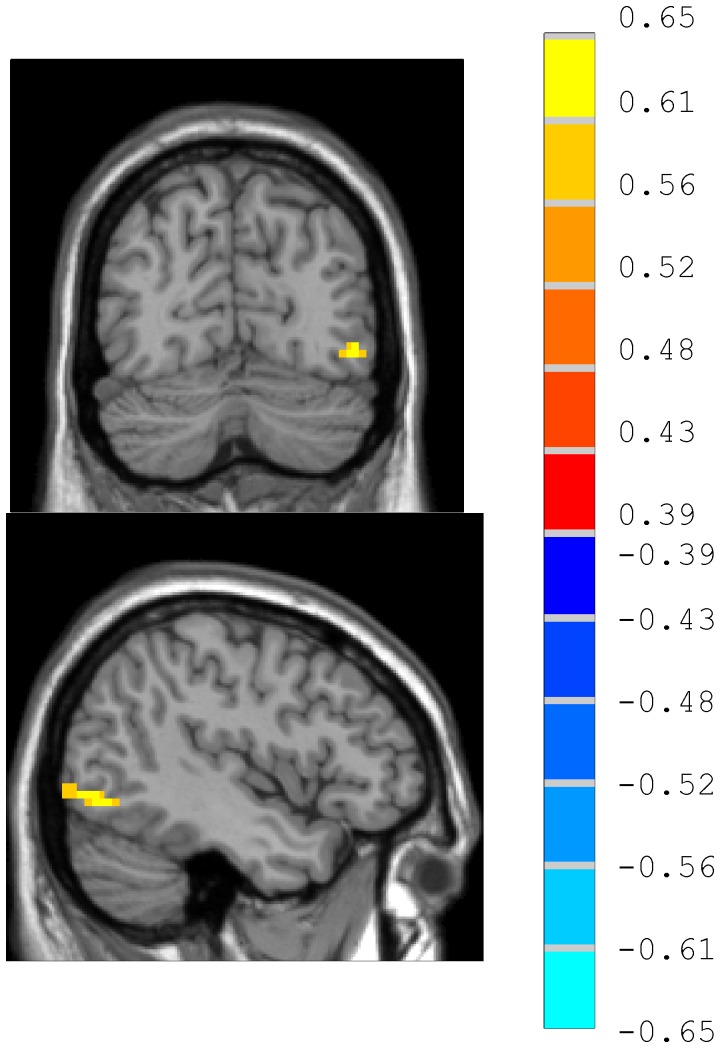
The identified brain region is seed region where ReHo correlated significantly with the Kinsey scale for sexual preference and also is one of the regions where ReHo differed between the homosexual and the heterosexual groups. The seed region has 145 voxels. P<0.05, corrected.

**Figure 5:**
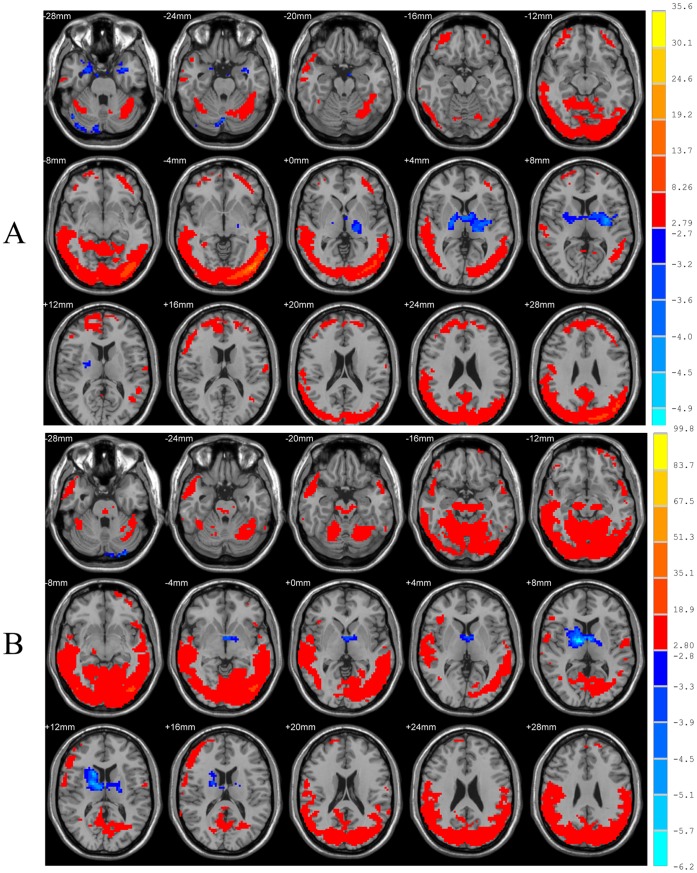
Functional connectivity in the resting state, shown as correlation maps of time series with the seed region in the homosexual group (A) and heterosexual group (B) (one-sample *t-*test; p<0.05, with multiple correction). T-sore bars are shown on the right. Hot and cold colors indicate higher or lower than the global mean, respectively.

Because we detected an inverse correlation between Kinsey Scale scores and ReHo of the left inferior occipital gyrus in the homosexual group, and because we also found that the left occipital gyrus was one of the brain regions where ReHo differed between the homosexual and the heterosexual groups, we selected the left inferior occipital gyrus as the seed region for FC analysis. The seed has 145 voxels and is showed in Figure 4. Activity in this seed region in the homosexual group positively correlated with activaty in the left angular gyrus, bilateral middle frontal gyrus, right anterior cingulate gyus, right superior frontal gurus, right cerebellum, left postcentral gyrus, right middle temporal gyrus, and left posterior cingulate gyrus. Activity of this seed region in the homosexual group also significantly inversely correlated with activity in bilateral cerebellum, bilateral cingulate gyrus, right parahippocampus, left middle temporal gyrus, and left putamen (one sample *t* test, p<0.05, corrected, Figure 5, Table 4).

**Table 4:** Brain regions showing significantly correlation with the left inferior occipital gyurs in homosexual group (one sample *t-*test, p<0.05, corrected).

Brain areas	Brodmann areas	Coordinates			t value	Cluster size
		x	y	z		
Left Inferiro Occipital Gyrus	18	−45	−78	−6	127.06	3898
Left Angular Gyrus	39	−45	−75	30	14.90	3282
Left Middle Frotal Gyrus		−33	48	−3	6.31	149
Right Anterior Cingulate	10	9	45	15	4.99	487
Right middle frontal gyrus	8	36	27	51	4.90	187
Right Superior Frontal Gyrus	11	24	54	−15	4.88	160
Cerebelum_6_R		30	−60	−27	4.40	53
Left Postcentral Gryrus	43	−63	−12	15	4.37	31
Right Middle Temporal Gyrus	21	57	3	−21	4.25	38
Right Middle Temporal Gyrus		66	−15	−24	4.04	27
Right Middle Frontal Gyrus		30	−9	63	3.92	25
Left Posterior Cingulate	30	−3	−57	6	3.39	24
Cerebelum_9_L		−3	−60	−54	−3.61	34
Cerebelum_Crus1_R		45	−69	−27	−3.71	26
Right cingulate gyrus		18	−9	39	−3.77	22
Left cingulate gyrus	24	−15	−18	39	−3.87	43
Vermis_7		3	−75	−24	−3.99	27
ParaHippocampal_R	28	24	3	−30	−4.39	149
Left Inferior Temporal Gyrus		−33	6	−36	−4.57	119
Left Putamen		−30	−12	6	−5.45	380

Activity in this seed region in the heterosexual group correlated positively with activity in the left inferior occipital gyrus, right middle temporal gyrus, right inferior frontal gyrus, right inferior frontal gryus, bilateral superior frontal gyrus, right fusiform gyrus, left middle frontal gyrus and right insula. Activity in this seed region in the heterosexual group also inversely correlated with activity in left parahippocampus, left cingulate gyrus, and right thalamus (one sample *t* test, p<0.05, corrected, Figure 5, Table 5).

**Table 5:** Brain regions showing significantly correlation with the left inferior occipital gyurs in heterosexual group (one sample *t-*test, P<0.05, corrected).

Brain areas	Brodmann areas	Coordinates			t value	Cluster size
		x	y	z		
Left Inferior Occipital Gyrus	18	−42	−78	−6	99.88	11349
Right Middle Temporal Gyrus		51	9	−18	5.79	174
Right Inferior Temporal Gyrus		54	−6	−42	5.37	39
Right Inferior Frontal Gyrus		51	30	15	4.98	146
Left Superior Frontal Gyrus		−33	21	54	4.87	125
Right Fusiform Gyrus		42	−42	−27	4.72	47
Right Inferior Frontal Gyrus		42	42	−18	4.38	22
Right Superior Frontal Gyrus		18	36	48	4.24	151
Left Superior Frontal Gyrus	11	−21	63	−12	4.28	20
Left Middle Frontal Gyrus		−33	51	−6	3.67	26
Right Superior Frontal Gyrus		9	57	30	3.64	21
Right Insula	45	39	24	3	3.54	18
Left ParaHippocampus		−24	0	−33	−3.47	19
Left Cingulate Gyrus		−15	3	36	−4.58	28
Right Thalamus		12	−6	9	−6.29	312

**Figure 6:**
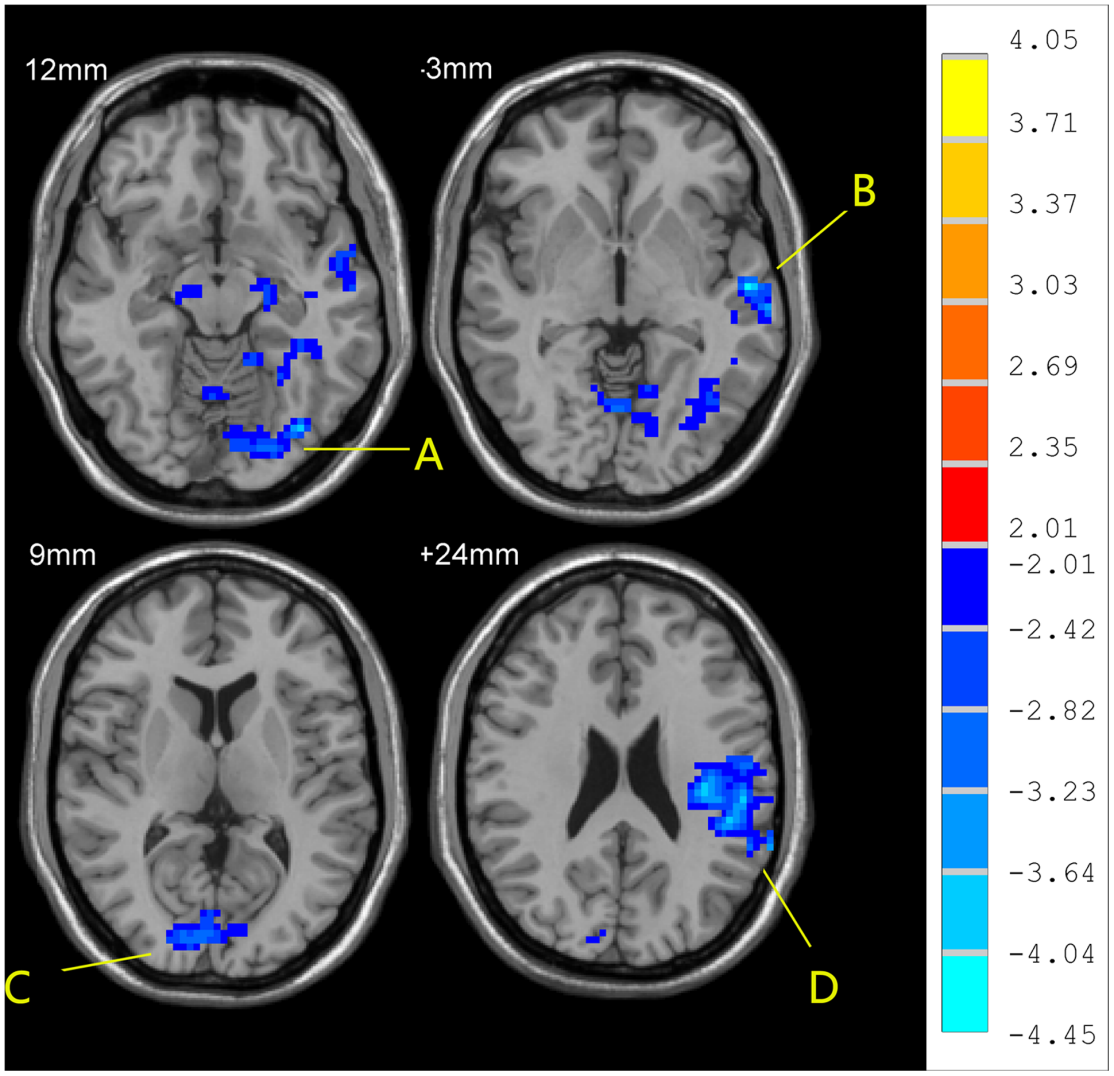
Differences in functional connectivity (differences in r-value maps) in the resting state between homosexual and heterosexual groups (two-sample *t*-test; p<0.05, corrected). T-score bars are shown on the right. Hot and cold colors indicate increased and decreased FC of homosexual group, respectively, compared with heterosexual group. The identified brain regions are the following: (A) left inferior occipital gyrus; (B) left middle temporal gyrus; (C) right cuneus; (D) left supramarginal gyrus.

Significant differences were noted in *z* value FC maps in the resting state between homosexual and heterosexual subjects. The homosexual participants showed decreased FC in left middle temporal gyrus, left supra-marginal gryus, left inferior occipital gyrus, right cuneus, compared with heterosexual subjects (two-sample *t* test, p<0.05, corrected; Figure 6, Table 6).

**Table 6:** Brain regions showing significant differences in functional connectivity between homosexual and heterosexual groups (two-sample *t-*test, p<0.05, corrected).

Brain areas	Brodmann areas	Coordinates			t value	Cluster size
		x	y	z		
Left Middle temporal Gyrus		−57	−15	−3	−4.45	165
Left SupraMarginal Gyurs		−48	−36	24	−3.86	234
Left Inferior Occipital Gyrus		−36	−78	−12	−3.75	578
Right Cuneus		9	−90	9	−3.22	159

### Correlation between FC and Kinsey Scale Scores in the Homosexual Group

**Figure 7:**
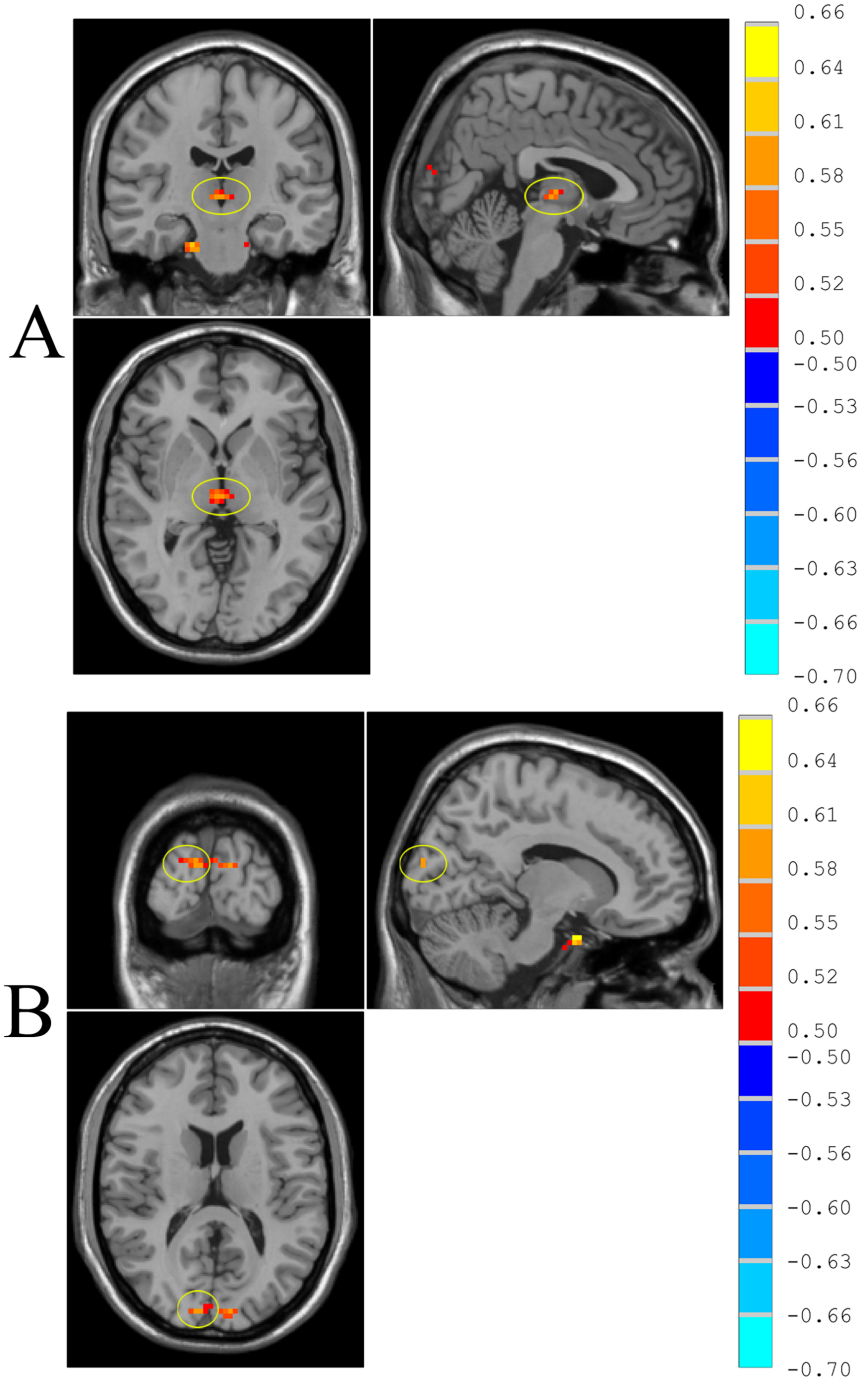
Significant correlations of FC in the resting state with Kinsey Scale scores within the homosexual group (correlation analysis, p<0.01, corrected). R-value are shown on the right. The identified region is right thalamus (A) and right cuneus (B).

When the seed was located in the left inferior occipital gyrus, significant positive correlations were observed between Kinsey Scale scores and *z* value maps from the FC analysis in the right cuneus and the right thalamus (P<0.01, corrected, Figure 7, Table 7).

**Table 7:** Brain regions showing significantly correlation between FC and Kinsey Scale scores (correlation analysis, p<0.01, corrected).

Brain areas	Brodmannareas	Coordinates			r value	Cluster size
		x	y	z		
Right Thalamus		3	−18	0	0.59	18
Right Cuneus	18	12	−93	15	0.59	22

## Discussion

Homosexual compared with heterosexual participants had increased ReHo in the rectus gyrus, bilateral midbrain, and left temporal lobe, and decreased ReHo in left inferior occipital gyrus, right middle occipital gyrus, right superior occipital gyrus, left cuneus, and right precuneus. In addition, ReHo in the left inferior occipital gyrus correlated with the Kinsey Scale scores. Using the left inferior occipital gyrus as the seeding area, homosexual participants showed decreased FC in left middle temporal gyrus, left supra-marginal gryus, left inferior occipital gyrus, and right cuneus. Additionally, FCs in right thalamus and right cuneus correlated positively with Kinsey Scale scores. To our knowledge, this is the first study focusing on the cerebral functions of homosexual men in resting state.

ReHo has been considered as a measure of the regional coherence of spontaneous BOLD fluctuations [23]. Unlike the functional connectivity that focuses on the long-distance interregional temporal correlations of BOLD signals, the ReHo targets identification of voxels where spontaneous activity is synchronous locally. Synchronized oscillatory activity in the cerebral cortex is thought to be essential for coordination and integration across space and time of activity in anatomically distributed but functionally related neural elements. High inter-regional synchronization of spontaneous activity in some brain regions could account for the increased efficiency of transferring information both within and across nodes in the network [24]. The investigation of ReHo in the resting state could help us to identify the functional characteristics of the brain at baseline, independent of confounding differences in strategy and response characteristics in homosexual men when performing a task.

ReHo in the left supplementary motor area correlated negatively with Kinsey scores, indicating that ReHo in exclusively homosexual men was reduced in the supplementary motor area. The supplementary motor area contributes to the ordering of sequential movements and the planning and coding of several movements ahead [25]. It is also participates in mental rotation [26], [27] and verbal fluency [28]. Several studies have reported that homosexual men have poorer mental rotation compared with heterosexual men [29]. Homosexual compared with heterosexual men have weaker spatial abilities relative to verbal fluency but similar to heterosexual women [30]. Based on correlation analysis of ReHo with Kinsey scores, we hypothesized that the reduced ReHo in the supplementary motor area could affect the ability of homosexual men to integrate spatial information and may account in part for their poorer spatial abilities. However, the difference of ReHo between homosexual and heterosexual groups in the supplementary motor area was not significant in the study. So functional characteristic of supplementary motor area in homosexual group need further study.

Moreover, ReHo in the left inferior occipital gyrus was reduced in homosexual men. The inferior occipital gyrus plays an important role in visuospatial processing [31], skills that demonstrate robust sex differences [32]–[34]. Men tend to score higher than women on tests of mental rotation, spatial perception, and orientation [35]–[37], whereas women tend to score higher on tests of memory for object locations in spatial arrays [38]. Homosexual men tend to show female-like performance on tests of mental rotation and judgment of line orientation tasks [39]. Fransson [40] found that monitoring of the environment, self-reflective thoughts, and emotional states are likely most active when participants are in the “resting state”, and monitoring of the environment employs visuospatial skills, suggesting that reduced ReHo in the left inferior occipital could be a consequence of group differences in deployment of visuospatial processing in the resting state. Additionally, in the homosexual group we found a positive correlation between ReHo in the left inferior occipital gyrus and the degree of homosexual preference (*r* = 0.53, *p*<0.05), indicating that this visuospatial processing activity may relate to the degree of homosexual orientation.

Functional connectivity analyses can help to identify differences in functional networks between male homosexuals and heterosexuals. We used the left inferior occipital gyrus as a seed region and compared FC across the two group. We found reduced FC in homosexual compared with heterosexual participants in the left middle temporal gyrus, left supra-marginal gryus, left inferior occipital gyrus, and right cuneus. The decreased FC in the left middle temporal gyrus may relate to the previously characterized verbal ability of homosexual men. The left middle temporal gyrus plays an important role in semantic judgments about words [41]. It has been hypothesized that language functions are more strongly lateralized to the left hemisphere in males than in females [42], [43]. In an fMRI study, comparing activation in the middle and superior gyrus during a story with activation during reverse replay of the story showed lateralization to the left hemisphere in males but not in females. Thus males seem to use the left posterior temporal lobes during linguistic processing, whereas females use temporal cortices more bilaterally [44]. In the present study, heterosexual participants showed increased FC in the left middle temporal gyrus, compared with homosexual participants, suggesting that semantic functions in homosexual persons might be based in part bilaterally in the middle temporal gyrus, a possibility supported by prior studies. Developmental instability of neurocognitive functions related to sexual orientation was found to exist only in men, but not in women [45]. It suggested that neurocognitive functions of brain in women had characteristic symmetry. In addition, Wegesin *et al.* [46] found that homosexual men performed akin to heterosexual women on the verbal and semantic monitoring tasks which was cognitive task for assessing verbal ability. So we hypothesized the decreased functional connectivity between left middle temporal gyrus and left inferior occipital gyrus might be a special marker for neurocognitive functioning in homosexual men.

On the other hand, inferior occipital gyrus is also considered as visual area V2 which is the second major area in the visual cortex and the fiirst region within the visual association area [47]. Middle temporal has a region, known as visual area V5, associated with visual function. Visual area V5 is a region of extrastriate visual cortex that is thought to play a major role in the perception of motion, the integration of local motion signals into global percepts and the guidance of some eye movements [48]. Middle temporal is connected to visual area V2. The first studies of the electrophysiological properties of neuros in middle temporal showed that a large portion of the cells were tuned to the speed and directionof moving visual stimuli. These results suggested that middle temporal played a significant role in the processing of visual motion [49], [50]. Several studies have showed that homosexual men had poor ability of visual and spatial orientation than that of heterosexual men [29], [39]. So the decrease of functional connectivity between left inferior occipital gyrus and left middle temporal gyrus may be associated with the poor visual and spatial ability of homosexual men.

Another important language area in the brain, the left supramarginal gyrus, also plays an important role in phonological processing [51]. Sex difference in cerebral laterality of phonological task was found in an fMRI study. Just the same as that in the semantic process, men showed left lateralized during the phonological task, however, women showed bilateral activity during the same task [52]. Decreased functional connectivity between left supra-marginal gyrus and left inferior occipital gyrus might be associated with female-like feature of phonological process for the homosexual participants.

We also found that the functional connectivity correlated positively with Kinsey Scale scores in the right thalamus (Figure 7) and right cuneus. The thalamus received visual information from the occipital gyrus and is involved in human sexual arousal. Kranz [11] found that the medial dorsal nucleus of the thalamus and medial orbitofrontal cortex activated more in heterosexual men and homosexual women viewing female faces in and in homosexual men and heterosexual women viewing male faces. Another fMRI study compared sexual arousal in homosexual and heterosexual people of both genders, using images of naked male or female torsos displaying signs of genital arousal, and found that the centromedian thalamus, ventral striatum, and ventral premotor cortex showed stronger neuronal responses to preferred relative to nonpreferred stimuli across groups [12].
