# Correction: Association of Cerebral Networks in Resting State with Sexual Preference of Homosexual Men: A Study of Regional Homogeneity and Functional Connectivity

**DOI:** 10.1371/annotation/79003c6c-f167-463d-81db-e16b55c7a2dd

**Published:** 2013-06-19

**Authors:** Shaohua Hu, Dongrong Xu, Bradley Peterson, Qidong Wang, Xiaofu He, Jianbo Hu, Xiaojun Xu, Ning Wei, Dan Long, Manli Huang, Weihua Zhou, Weijuan Xu, Minming Zhang, Yi Xu

The name of the third author is incorrect. The correct name is: Bradley S. Peterson. The correct citation is: Hu S, Xu D, Peterson BS, Wang Q, He X, et al. (2013) Association of Cerebral Networks in Resting State with Sexual Preference of Homosexual Men: A Study of Regional Homogeneity and Functional Connectivity. PLoS ONE 8(3): e59426. The correct abbreviation in Contributions is: BSP. 

